# Potential Effects of Hydroelectric Dam Development in the Mekong River Basin on the Migration of Siamese Mud Carp (*Henicorhynchus siamensis* and *H. lobatus*) Elucidated by Otolith Microchemistry

**DOI:** 10.1371/journal.pone.0103722

**Published:** 2014-08-06

**Authors:** Michio Fukushima, Tuantong Jutagate, Chaiwut Grudpan, Pisit Phomikong, Seiichi Nohara

**Affiliations:** 1 National Institute for Environmental Studies, Tsukuba, Japan; 2 Department of Fisheries, Faculty of Agriculture, Ubon Ratchathani University, Ubon Ratchathani, Thailand; Pacific Northwest National Laboratory, United States of America

## Abstract

The migration of Siamese mud carp (*Henicorhynchus siamensis* and *H. lobatus*), two of the most economically important fish species in the Mekong River, was studied using an otolith microchemistry technique. Fish and river water samples were collected in seven regions throughout the whole basin in Thailand, Laos and Cambodia over a 4 year study period. There was coherence between the elements in the ambient water and on the surface of the otoliths, with strontium (Sr) and barium (Ba) showing the strongest correlation. The partition coefficients were 0.409–0.496 for Sr and 0.055 for Ba. Otolith Sr-Ba profiles indicated extensive synchronized migrations with similar natal origins among individuals within the same region. *H. siamensis* movement has been severely suppressed in a tributary system where a series of irrigation dams has blocked their migration. *H. lobatus* collected both below and above the Khone Falls in the mainstream Mekong exhibited statistically different otolith surface elemental signatures but similar core elemental signatures. This result suggests a population originating from a single natal origin but bypassing the waterfalls through a passable side channel where a major hydroelectric dam is planned. The potential effects of damming in the Mekong River are discussed.

## Introduction

Migratory behaviors are extremely common in fisheries, and understanding fish migrations is vital to sustainable management and conservation of fisheries resources. While diadromous species and populations have long been studied mainly in temperate regions, potamodromous migrations that are common in large tropical rivers, such as the Amazon and Mekong Rivers are much less understood [1]–[4].

The Mekong River, an international river rising in China and flowing 4,200 km through five other countries of Southeast Asia, is renowned as the world's largest inland fishery, as well as for harboring the second most diverse fish fauna after the Amazon River [5]. It produces over 2 million tonnes of wild fish and other aquatic animals each year within its catchment [6] and supports more than 1,200 fish species, although many have not yet been described [7].

There is a clear and urgent need to better understand fish movements for two main reasons. Firstly, a significant proportion of Mekong fishes are migratory, specifically potamodromous (i.e., migratory wholly in freshwater) [8], and, more importantly, this enormous fisheries production relies heavily on the migratory habit of these species [9], [10]. They are harvested mostly with gears and techniques developed to capture fishes during their seasonal migrations [11]–[14]. Secondly, the Mekong fishes and fisheries now face an imminent threat from massive hydropower development. Currently blocked by mainstream dams only in the uppermost Chinese territory, the Mekong River has 11 more dam proposals on the mainstream and more than 100 in tributary systems in the lower basin, all of which are scheduled to be installed within the next decade [15]. These dams will impact fishes directly as a barrier to their migrations or indirectly through creation of huge impoundments and alterations to the natural flow regime, leading to a substantial loss of fish species and their habitats [10], [16]–[18].

Of the vast multitude of fish species in the Mekong River, two closely-related species, *Henicorhynchus siamensis* and *H. lobatus*, are of special concern given the rapid rate of hydropower development. Inhabiting the Mekong and Chao Phraya basins (*H. siamensis*) and the Mekong basin (*H. lobatus*), these two small-sized cyprinids, collectively referred to as Siamese mud carp, are the most abundant and most economically important fish in the middle and lower Mekong basin [19], [20]. They are harvested in huge numbers, especially in and around Tonle Sap Lake in Cambodia and the Khone Falls area in southern Laos. These species account for 43% [11], [14] and over 50% [13] of the total catch in these areas, respectively, with an overall basin-wide catch being >12% for the two species combined [5].

The Siamese mud carp populations in the Mekong, or at least some of them, are known to perform long-distance migrations [4], [7], [12]. However, what is known about their migrations is severely limited. In Thailand and Laos, the species undertake upstream migration in the early rainy season above the Khone Falls, whereas in Cambodia, this species migrate upstream at the onset of the dry season below the waterfalls [4]. The latter migrants possibly originate from the Tonle Sap Lake, first descending the Tonle Sap River and then ascending the Mekong mainstream toward and even past the Khone Falls [12]. Spawning takes place in tributaries or floodplains during the wet season, with a peak in May-June [21]. Eggs and larvae are carried to nursery habitats on the floodplain by the water current [4]. At the beginning of the dry season, juveniles move out of the floodplains with the receding water and seek dry season refuge habitats such as deep pools in the Mekong mainstream.

Multiple genetically discrete populations or stocks have been identified in both species of Siamese mud carp within the Mekong basin [22], [23]. However, ecological evidence that supports the genetic differentiation other than the single natural barrier of the Khone Falls has been lacking, thus hampering the development of effective fisheries management strategies.

We used an otolith microchemistry technique to elucidate the natal origins and subsequent movement patterns of the Siamese mud carp in the Mekong River. Otoliths (ear bones) are paired calcified structures that continually grow by accreting calcium as well as other elements from ambient waters onto their surface [24]. Because of acellular and metabolically inert properties, the otoliths provide a chemical chronology over the entire life of a fish [25]. Although otolith microchemistry was originally developed for marine or diadromous fishes that are exposed to higher elemental concentrations than freshwater fishes or undergo a substantial change in elemental concentrations during their life histories, the technique has increasingly been applied to obligate freshwater species. For example, to locate their natal origins [26]–[28] or maternal origins [29], to elucidate migration patterns [30]–[32], to discriminate wild from hatchery populations [33], and to detect exposure to pollutants from mine tailings [34]. Based on extensive field sampling throughout the lower Mekong basin, we compared the migration patterns of the Siamese mud carp between species as well as among geographical regions with particular emphasis on predicting the potential impacts of the proposed hydropower projects on these highly valuable fisheries resources.

## Materials and Methods

### Study area

**Figure 1:**
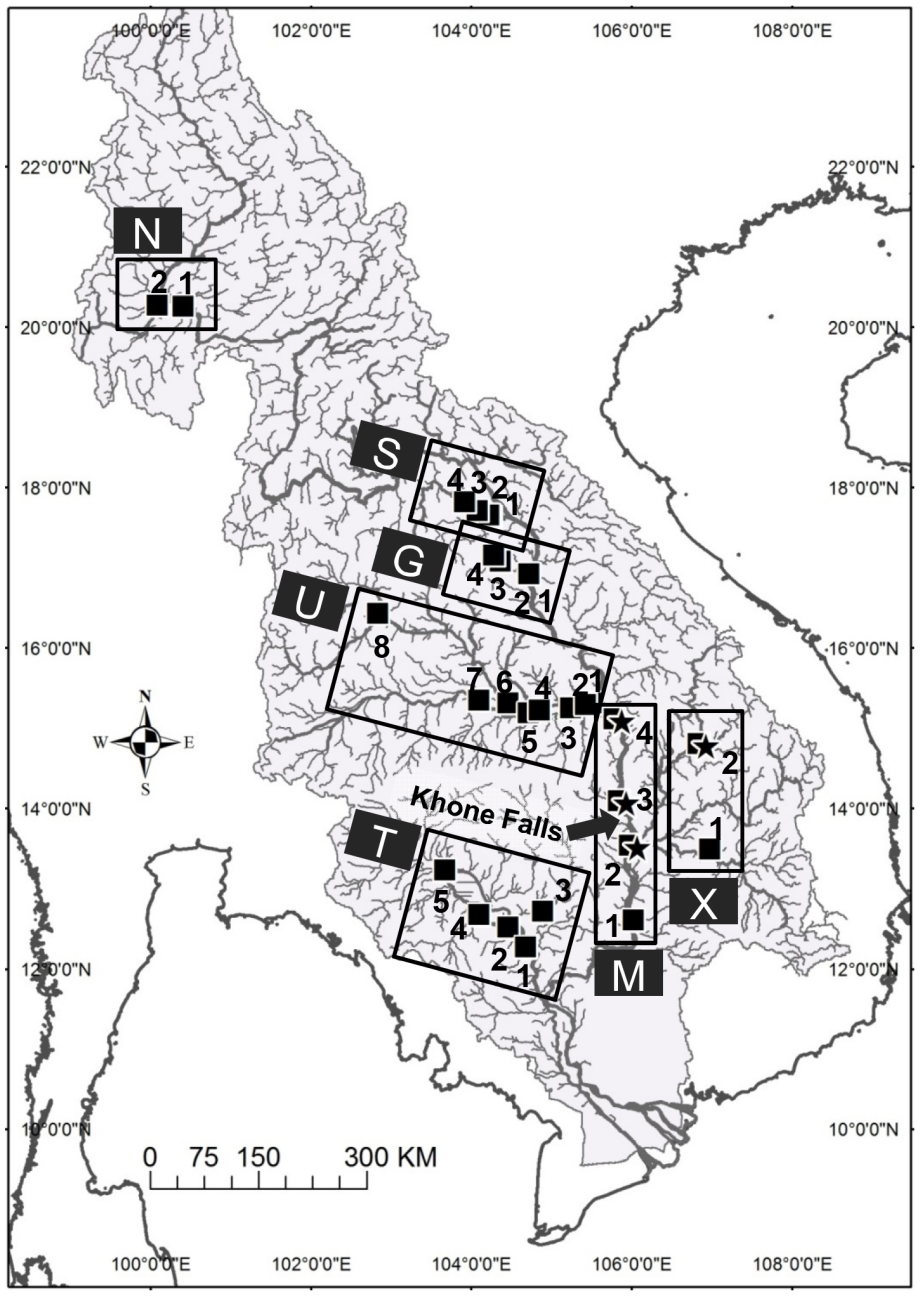
Fish sampling sites in the Mekong River basin. Black square: *Henicorhynchus siamensis*. Star: *H. lobatus*. A total of 29 sites are serially numbered toward upstream within each of seven regions: N =  Thai North, S =  Songkhram, G =  Gam, U =  Mun, X =  Xekong, T =  Tonle Sap and M =  Mekong mainstream (MM). Location of the Khone Falls is indicated.

The study was conducted in the lower Mekong basin of Thailand, Laos and Cambodia (Fig. 1). The study area ranged in elevation from about 10 m a.s.l. at Kampong Chhnang, Cambodia (T1) to 350 m a.s.l. at Chiang Saen, Thailand (N2). River discharge generally peaks in September, decreases toward the end of the dry season in April and starts to increase in May. Fish sampling was conducted during 2007–2010 at the total of 29 sites in the mainstream and tributary systems in the following seven regions: the Mekong River in the Thai North (N1-2), the Songkhram (S1-4), Gam (G1-4), Mun (U1-8), and Xekong Rivers (X1-2), Tonle Sap Lake (T1-5) and the Mekong mainstream in southern Laos and Cambodia (M1-4, hereafter referred to as MM).

Immediately below M3 in southern Laos near the Cambodian border are located the Khone Falls, the only natural waterfalls in the Mekong River that diverge into series of cataracts across the river width. Despite the large elevation gap of 21 m, one side channel called Hoo Sahong bypasses the waterfalls without any natural barriers along its approximately 7 km length, providing the single most important fish passage route year-round for the migratory fish species of the Mekong River [12], [35].

Most of the tributaries sampled were freely accessible from the Mekong River except for the Gam and Mun Rivers. In the Gam River, there are five irrigation dams located with approximately equal spacing along its 100-km long course before emptying into the Mekong River [36]. The most recent of the five dams was constructed in 2009 in the lowermost reach of the Gam, about 2 km from the Mekong confluence. In the Mun River, a large hydroelectric dam, Pak Mun dam (17 m high; 136 MW), has blocked river water and possibly fish migrations at about 6 km from the Mekong confluence since 1994. The sluice gates of the dam have been kept open during the rainy season each year to allow for fish passage to mitigate adverse impacts on local fisheries [37], [38].

Several individuals of *Henicorhynchus siamensis and H. lobatus* were purchased at a fish landing site or the nearby local market, ensuring that the fish were captured in the vicinity and fresh (a few hours or less after landing). All the fish were measured for fork length to the nearest millimeter and weighed to the nearest gram.

### Chemical analysis of otoliths

A pair of otoliths (lapilli) of each fish was carefully extracted using ceramic forceps within a few hours of fish sampling. In Ostariophysi species such as cyprinids, lapilli are preferred otoliths to analyze because they are the largest of the three otoliths of a fish, have the single leading growth axis, and undergo conservative shape change throughout life [39]. The otoliths were immediately stored in a 2-ml polypropylene tube separately for each fish for transportation to the National Institute for Environmental Studies, Japan. In the laboratory, the otoliths were submerged into 30% hydrogen peroxide for 30 minutes and moved to a tube filled with ultrapure water (Milli-Q) for ultrasonic washing for 5 minutes to remove soft tissue around the otoliths. The otoliths were then triple rinsed with Milli-Q water and air dried in a clean booth equipped with HEPA filters. One of the pairs of lapilli was mounted whole on a glass slide with sulcus side down using thermoplastic glue (Crystalbond) for otolith surface assays. The other lapillus was embedded with high-transparency polyester resin (Technovit 7200VLC, Heraeus Kulzer). After complete polymerization under fluorescent light for about 24 h, the embedded lapillus was sliced into a thin section in the frontal plane using a low-speed diamond wheel saw (SBT650, South Bay Technology). The sectioned sample was then mounted on a glass slide using Crystalbond for grinding with sandpaper and polishing with diamond slurry of 9 and then 3 µm to a thickness of 150–200 µm until the otolith core (i.e., primordium) was exposed. The mounted sectioned sample was then sonicated, rinsed with Mill-Q to remove grinding particles and air dried. Both whole and sectioned otolith samples were kept in the clean booth until analysis. The sectioned samples were assayed only when greater than or equal to five otoliths were obtained at a given site-date combination.

We used laser ablation inductively coupled plasma mass spectrometry (LA-ICP-MS; Agilent 7500cx ICP-MS, coupled with New Wave Research UP213 system equipped with a Nd:YAG 213-nm laser) to assay ^23^Na, ^24^Mg, ^44^Ca, ^55^Mn, ^66^Zn, ^88^Sr, and ^137^Ba in the otolith samples. Otolith calcium (^44^Ca) was used as an internal reference to correct for any changes in materials ablated from sampling location to sampling location. A standard material produced by the National Institute of Standards and Technology (NIST 612) was used as an external reference to adjust for possible instrumental drift as explained below. Laser parameters were 80–100% laser power and 20 Hz repetition rate with a dwell time of 2 s per spot for both whole and sectioned samples.

For whole otolith samples, a total of five spots of 90-µm diameter were ablated by directly applying the laser beam perpendicularly to the otolith surface to determine average elemental concentrations. For sectioned otolith samples, a series of 55-µm diameter spots each separated by 60 µm were ablated along the core-to-edge transect of the longest axis; on average 24±3 (mean ± SD) spots were ablated depending on the otolith size. Data from ablation spots that overlapped, even partially, with surrounding embedding material near the otolith edge, were excluded from further analyses. Finally the otolith surface elemental data were combined with the transect data for each otolith by adding the former to the last (i.e., edge) data point of the latter, producing a complete otolith profile of the fish's birth-to-death chemical history. Despite different ablation techniques adopted, the surface data were smoothly connected to the transect data without any noticeable gaps.

The assay of a batch of five otoliths were preceded and followed by those of the NIST glass for calibration [40]. All LA-ICP-MS analyses involved background data collection for 40 s, followed by otolith data collection for 60 s per spot. Helium gas stream was used to carry ablated materials from the laser system to the ICP-MS where it was mixed with Ar gas. Analytical accuracy of elements measured as the mean percent relative standard deviation (RSD) based on the NIST glass analyses were 8.3% (Na), 13.3% (Mg), 9.0% (Mn), 24.1% (Zn), 6.6% (Sr), and 7.3% (Ba). Limits of detection (LOD) were calculated for each element in each otolith sample as the blank value plus 3SD of the blank signals during the background data collection. Percentages of the samples that exceeded the LODs were 99.0/100 (Na), 100/100 (Mg), 80.7/72.35 (Mn), 99.0/89.9 (Zn), 100/100 (Sr), and 100/100 (Ba) for whole/sectioned samples.

### Water sampling and chemical analysis

**Figure 2:**
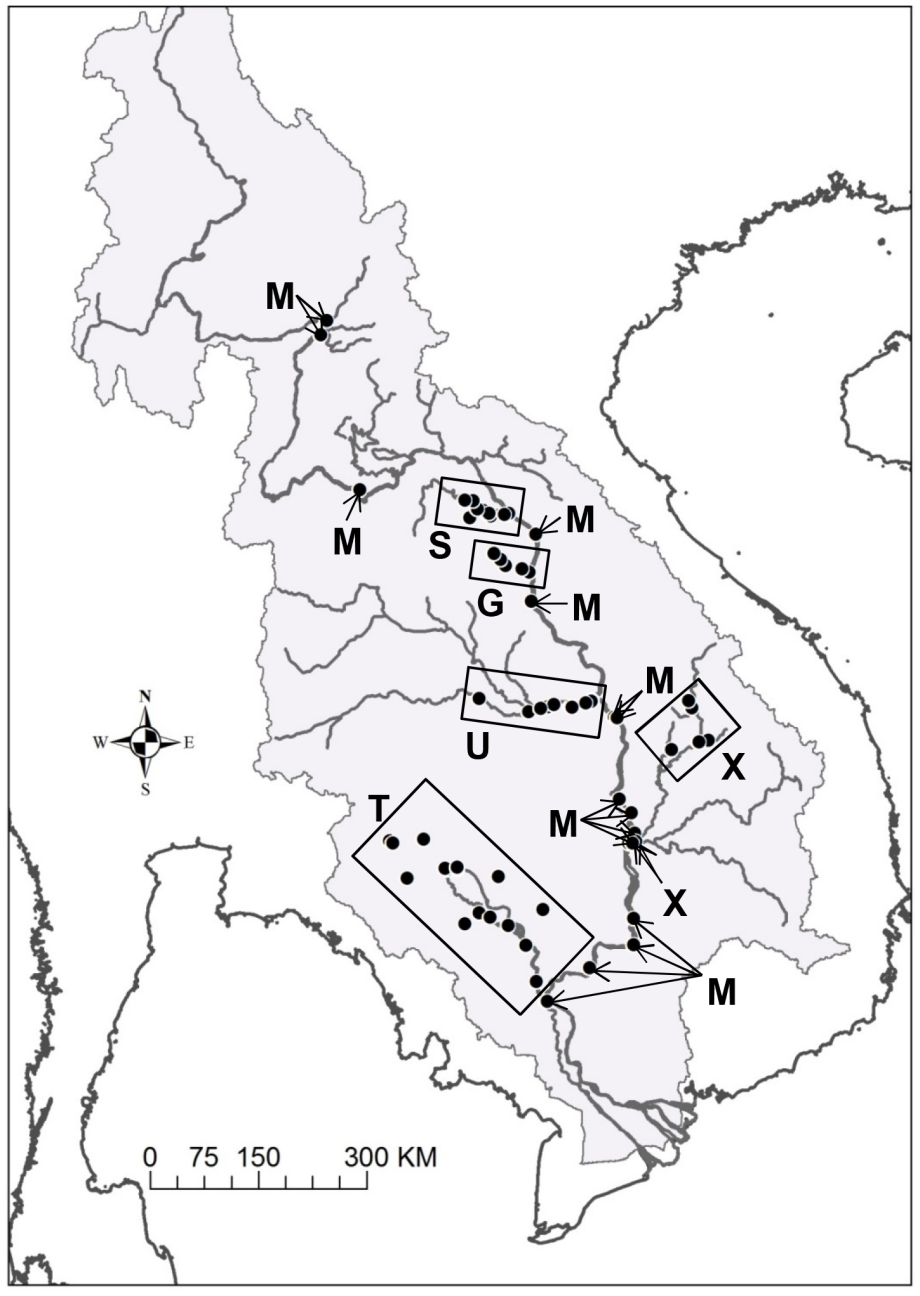
Water sampling sites in six regions. S =  Songkhram, G =  Gam, U =  Mun, X =  Xekong, T =  Tonle Sap and M =  Mekong mainstream.

River water was sampled at a total of 74 sites throughout the lower Mekong basin by encompassing all regions but the Thai North where only otoliths were sampled (Fig. 2). Water was collected at the same or closest site to a fish sampling site when the fish were bought far from the river. The sampling was conducted either from a boat or a bridge to avoid the direct influence of residential effluent alongside the river bank. Additional water samples were collected from up- and downstream of the original water sampling site when the confluences of large tributary systems existed in the vicinity to account for all potential water sources to which fish could have been exposed. The water samples were repeatedly collected in some regions to assess seasonal patterns in elemental signatures. Due to difficulties in accessing a river, however, samples could not be taken from some fish sampling sites in regions other than the Thai North. Conversely, the Mekong mainstream was extensively sampled for water from nearly as north as the Thai North to the Tonle Sap River confluence to the south. Immediately after the sampling, the water (14.5 ml) was filtered with 0.20 µm syringe filter into an acid-washed 15 ml polystyrene test tube to remove microorganisms and particles, acidified with 0.5 ml of aristar grade nitric acid to 1% v/v, and refrigerated until analysis.

The water samples were analyzed for the concentrations of the same set of elements as otoliths using both an Inductively Coupled Plasma Atomic Emission Spectrometer (ICP-AES) (ICAP-750, Nippon Jerrell-Ash Co. Ltd.) primarily for major and minor elements and an ICP-MS (Agilent 7500cx) for trace elements. Some elements were assayed by both instruments, but when ICP-MS readings exceeded 30 ppb, corresponding values from ICP-AES were adopted. The detection limits (ppb) of ICP-AES were 100 (Na), 20 (Mg), and 30 (Ca), and those of ICP-MS were 0.001 (Mn), 0.004 (Zn), 0.001 (Sr), and 0.003 (Ba) with all sample readings being above the detection limits. Elemental concentrations both in water and otoliths were converted to molar ratios of analyte/Ca in all statistical analyses.

### Data analyses

Molar ratios of Mg, Mn, and Zn to Ca for both water and otolith samples were logarithmically transformed to achieve normality before statistical analyses. Na was excluded from all the analyses except water-otolith linear regression analysis because of its significant multicollinearity with most of the other elements.

To eliminate random noise from the otolith profile data of elements from the core to surface, the Gaussian-type 5-point running weighted average was applied to smooth the original data with a weight being equal to:
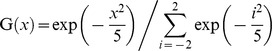
where an integer *x* varied from −2 to 2. This resulted in the losses of 2 consecutive original data points (or 120 µm) each from the core and edge portions of the profile, although the lost data were partially reflected in the first and second data points of the core portion as well as the last and second last points of the edge portion in the resultant smoothed profile.

Water sampling sites located within a 10-km radius of each fish sampling site were identified using the geographical information system (ArcGIS, ESRI) to calculate average water elemental concentrations for the fish site. Linear regression analyses were performed to examine relationships between water and otolith surface elements. The effects of fish size and sampling season on the water-otolith relationships were tested by incorporating fish size to the linear regression as a candidate explanatory variable and by constructing nested regression models with separate slopes and intercepts between wet (June-November) and dry (December-May) seasons, respectively. A partition coefficient of elements was estimated as a slope of a regression line through the origin.

Regional and temporal differences in water and otolith surface elemental signatures were tested with the two-way analysis of variance (ANOVA). Multivariate analysis of variance (MANOVA) was also performed with Pillai's trace statistics to compare otolith elemental signatures among regions and between species. Multiple comparisons among regions were made for selected elements using the Tukey's HSD test separately for otolith core and surface. Linear discriminant analyses (LDA) were applied separately to the water and otolith data to examine classification performance of the sampling regions. For the otolith LDA, only *H. siamensis* samples from all regions but the Xekong River, were used due to the limited samples available from this region. Elemental molar ratios were standardized to have the mean 0 and standard deviation 1 to allow the comparison of relative contributions by each element in the LDA. Jackknife cross-validation procedure determined classification accuracy of the regions. All the statistical analyses were conducted with a significance level of α = 0.05 using S-PLUS Version 8.1 (TIBCO Software Inc.).

### Ethics statement

Field sampling was carried out with the relevant permissions from Department of Fisheries, Thailand, Inland Fisheries Research and Development Institute, Cambodia, and Living Aquatic Resources Research Centre, Laos. This study did not involve any endangered or protected species. The species involved were originally collected by fishermen for human consumption using the approved fishing gear in respective countries. Therefore, no approval was obtained from any animal ethics committees.

## Results

### Water elemental signatures

**Figure 3:**
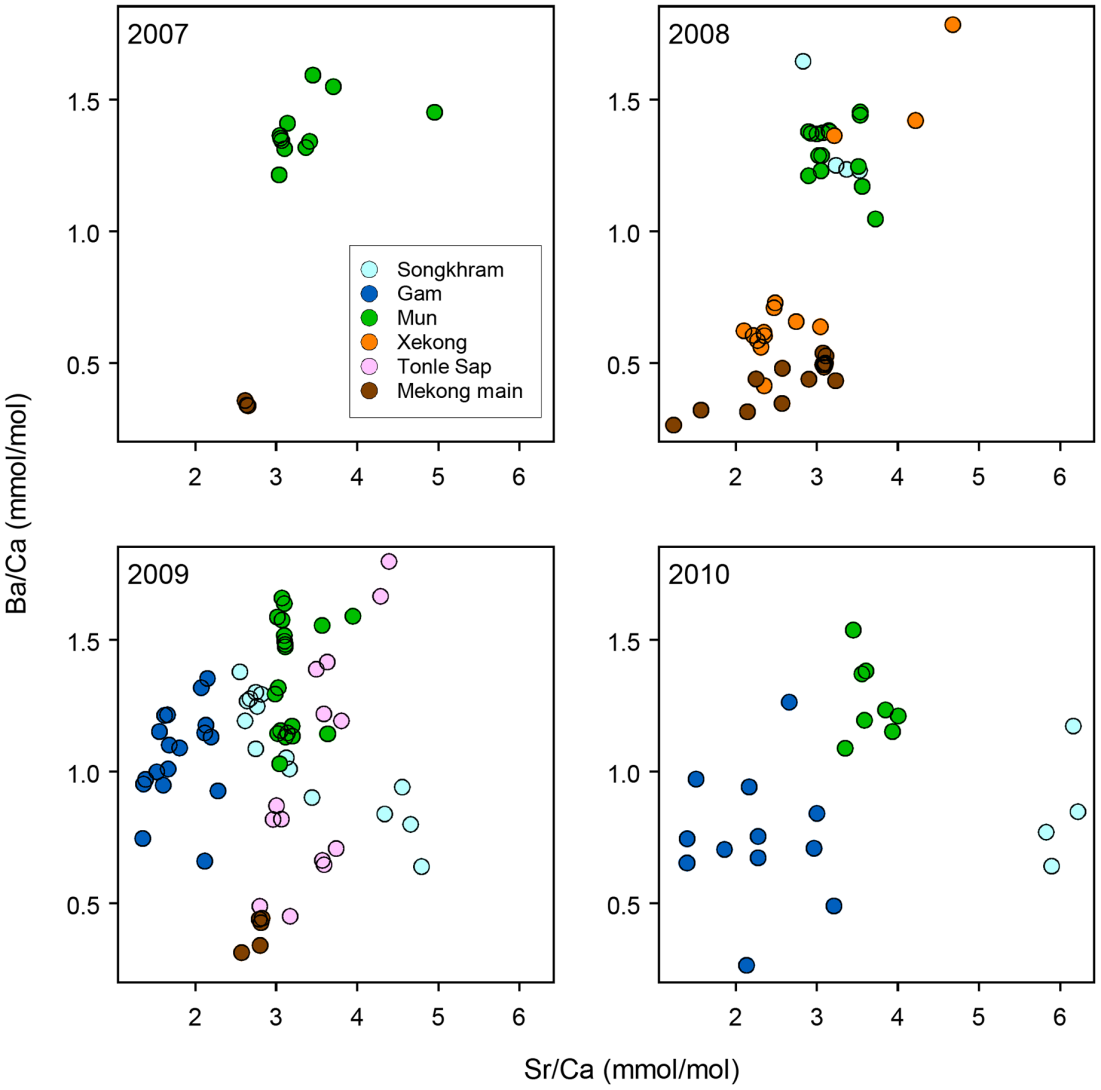
Sr-Ba elemental signatures of river water samples collected from six regions during 2007-2010.

A total of 158 water samples were collected throughout the lower Mekong basin (Table S1). Water elemental signatures differed among regions in each of five elements (i.e., Mg, Mn, Zn, Sr and Ba; p<0.05) and among years in Mg, Sr and Ba (p<0.05). To a large extent, the six regions were separated with two elements, i.e., Sr and Ba (Fig. 3). The LDA of the two-element model classified 68.4% of the data correctly to the regions of sampling, whereas a full model based on the five elements classified 86.7% of the data (Table 1). The correct classification rates were particularly high for the Mekong mainstream (100%) despite its extensive sampling area and the Mun River (98%), for which the percentages were not different between the models. Although the Songkhram River appears to have shifted its water Sr-Ba signatures over the years, the other regions were relatively consistent in the chemical signatures.

**Table 1:** A misclassification table of water samples by linear discriminant function analysis.

	Songkhram	Gam	Mun	Xekong	Tonle Sap	Mekong	% correct
Songkhram	**20**	1	0	0	2	0	87
Gam	0	**27**	0	0	1	2	90
Mun	1	0	**53**	0	0	0	98
Xekong	0	2	0	**10**	2	0	71
Tonle Sap	2	2	2	3	**4**	1	29
Mekong	0	0	0	0	0	**23**	100

Correct classifications are in bold, for which percentages are also given.

### Water-otolith relationships

**Figure 4:**
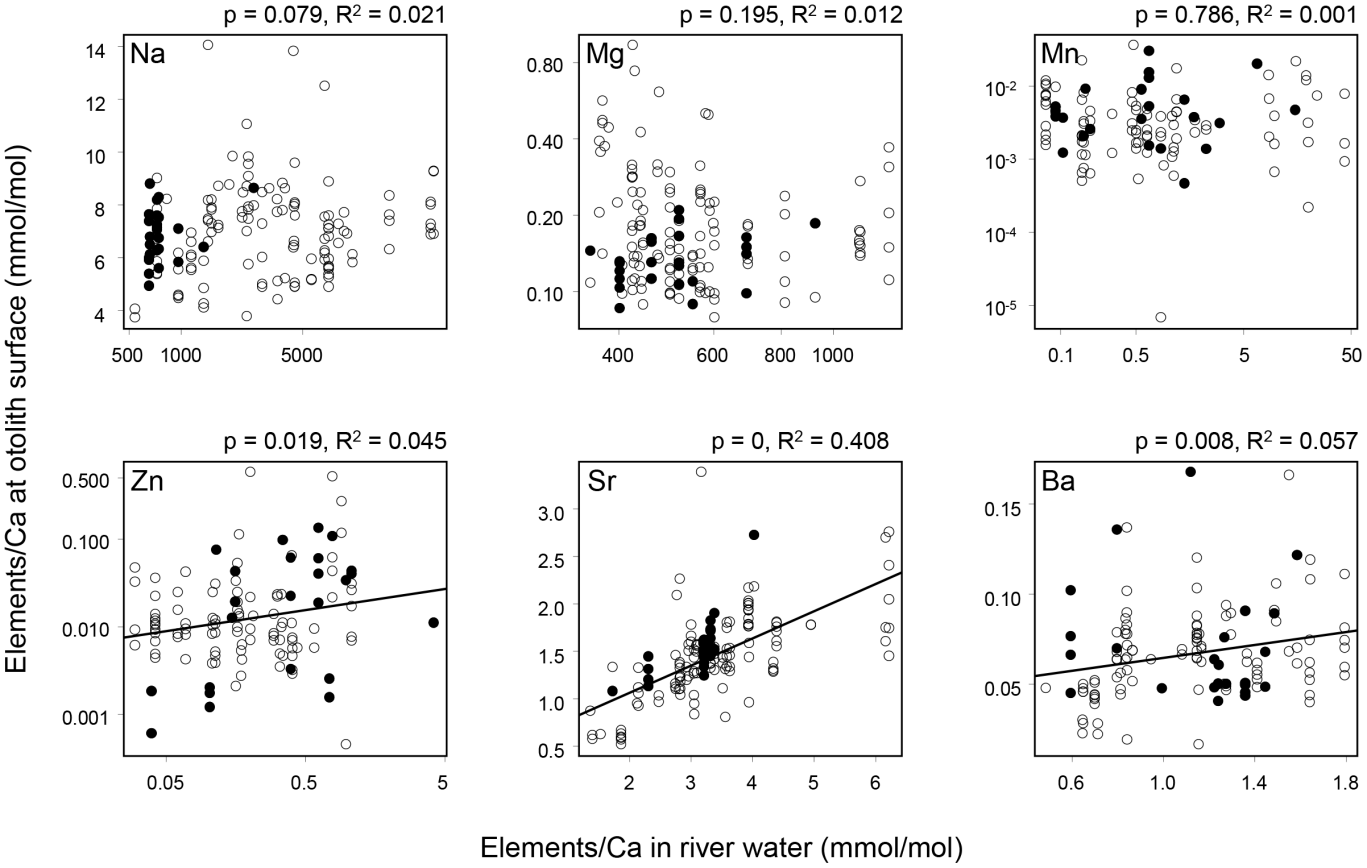
Otolith surface elemental signatures plotted against river water signatures. Open circle: *H. siamensis*. Closed circle: *H. lobatus*. Linear regression lines based on combined data from both species are superimposed, for which statistical significance and the coefficient of determination are shown above each panel.

A total of 173 *Henicorhynchus siamensis* and 24 *H. lobatus* were collected from the seven regions of the Mekong basin (Table S2). Fish fork length varied both among regions and sampling months with the overall mean ± SD of 131±22 mm for *H. siamensis* and 130±15 mm for *H. lobatus*. Water samples collected within a 10-km radius of fish sampling sites were available for 122 *H. siamensis* and all the 24 *H. lobatus* otolith samples. Linear regression lines between water and *H. siamensis* otolith surface elements were significant and positive for Zn, Sr and Ba, while for *H. lobatus* the only significant and positive regression was found for Sr (Fig. 4). Two regression lines for Sr were not different between the species both in intercepts and slopes (p>0.05). Partition coefficients were 0.043 for Zn (p = 0.001), 0.409 for Sr (p<0.001) and 0.055 for Ba (p<0.001) for *H. siamensis*, and 0.496 for Sr (p<0.001) for *H. lobatus*. Fish size had no significant effect on any of the water-otolith elemental relationships (p>0.05). However, the sampling season had a significant effect on Zn and Ba, such that only the otoliths and river water collected during the wet season (i.e., June-November) had significantly positive correlations (p = 0.001 and <0.001, respectively).

### Otolith elemental profiles

Of the five elements in *H. siamensis* otolith surfaces, Mg, Mn, Sr and Ba were different among regions (Mg: *F*_5, 160_ = 5.183, p<0.001; Mn: *F*_5, 160_ = 5.864, p<0.001, Sr: *F*_5, 160_ = 27.300, p<0.001; Ba: *F*_5, 160_ = 4.353, p = 0.001), and Mn, Sr and Ba were different among years as well (Mn: *F*_3, 160_ = 2.942, p = 0.035, Sr: *F*_3, 160_ = 7.871, p<0.001, Ba: *F*_3, 160_ = 6.352, p<0.001). The classification accuracy of the two-element (Sr and Ba) LDA model was low (39.1%), and that of the four-element model was still not very high (50.3%, Table 2). Note, however, that 17 out of 21 otoliths (81%) from the Gam River were correctly classified.

**Table 2:** A misclassification table of *H. siamensis* otolith samples by linear discriminant function analysis.

	Thai North	Songkhram	Gam	Mun	Tonle Sap	MM	% correct
Thai North	**5**	0	2	3	0	0	50
Songkhram	1	**3**	3	25	3	2	8
Gam	1	0	**17**	2	0	1	81
Mun	3	5	1	**37**	3	3	71
Tonle Sap	0	0	0	7	**14**	1	64
MM	3	1	0	9	4	**10**	37

Correct classifications are in bold, for which percentages are also given.

**Figure 5:**
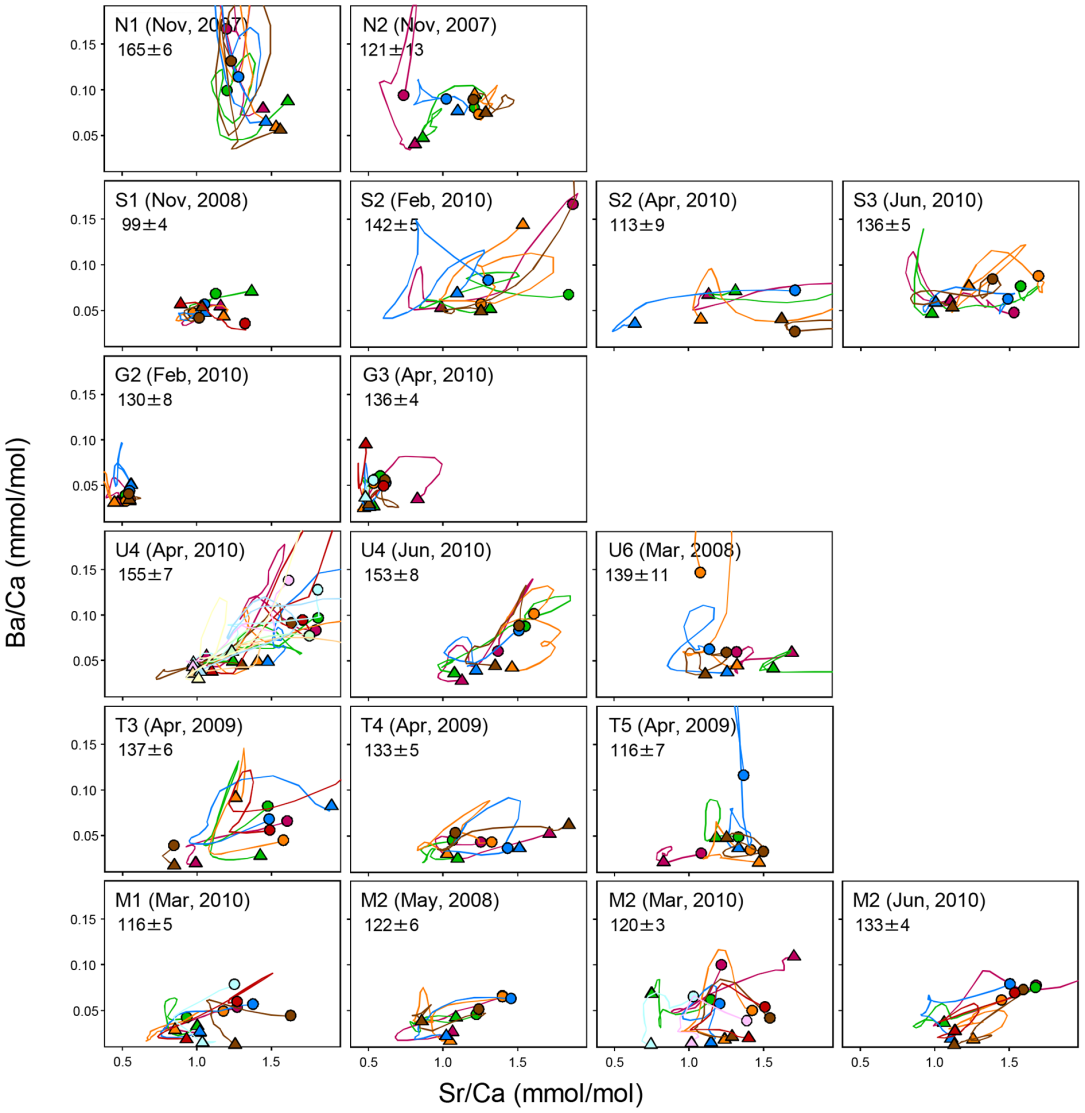
Sr-Ba elemental profiles along *H. siamensis* otolith transect. Triangle: otolith core. Circle: otolith surface. From top to bottom, otolith samples from Thai North (2), Songkhram (3), Gam (2), Mun (2), Tonle Sap (3), and Mekong mainstream (2) were analyzed with the number of sites in a parenthesis. Within a given site, different colors denote different individuals.

Otolith Sr and Ba for *H. siamensis* varied largely throughout life, but the shape of the elemental profiles resembled one another among individuals in a given site and even a region (Fig. 5). Between regions, however, otolith surface elemental signatures were different (Pillai's trace  = 0.814, *F*_10, 202_ = 13.872, p<0.001), and core signatures were also different (Pillai's trace  = 0.765, *F*_10, 202_ = 12.520, p<0.001). Otolith surface Sr signatures were different in all pairs of regions involving the Thai North and Gam River (Table 3). In particular, the Gam River exhibited the lowest level of otolith surface Sr among all regions. Otolith surface Ba was similarly different in many pairs involving these two regions. The otolith core Sr level was significantly lower again in the Gam River than the other regions (Table 4), and to a lesser extent the Ba level was also low in this region. The otolith core Ba was highest in the Thai North among all regions.

**Table 3:** Pairwise differences (mmol/mol) between regions in *H. siamensis* otolith surface elemental signatures for Sr/Ca (above diagonal) and Ba/Ca (below diagonal).

	Thai North	Songkhram	Gam	Mun	Tonle Sap	MM
Thai North		**−0.499**	**0.448**	**−0.716**	**−0.557**	**−0.395**
Songkhram	**−0.033**		**0.948**	−0.217	−0.058	0.104
Gam	**−0.055**	−0.022		**−1.16**	**−1.01**	**−0.844**
Mun	−0.021	0.012	**0.034**		0.159	0.321
Tonle Sap	**−0.038**	−0.005	0.017	−0.017		0.162
MM	**−0.029**	0.004	**0.026**	−0.008	0.009	

Significant differences are in bold. Values are average elemental molar ratios for regions in the row minus those in the column headings.

**Table 4:** Pairwise differences (mmol/mol) between regions in *H. siamensis* otolith core elemental signatures for Sr/Ca (above diagonal) and Ba/Ca (below diagonal).

	Thai North	Songkhram	Gam	Mun	Tonle Sap	MM
Thai North		0.141	**0.757**	0.063	−0.071	0.166
Songkhram	−0.009		**0.616**	−0.079	−0.212	0.024
Gam	−**0.030**	−0.022		−**0.694**	−**0.828**	−**0.592**
Mun	−**0.025**	−0.016	0.005		−0.134	0.103
Tonle Sap	−0.021	−0.012	0.009	0.004		0.236
MM	−**0.038**	−**0.029**	−0.008	−0.013	−0.017	

Significant differences are in bold. Values are average elemental molar ratios for regions in the row minus those in the column headings.

Fish from S1 exhibited little variations in both elements possibly due to their small body size (<100 mm). Despite large body sizes (≥130 mm), however, the Gam River fish showed severely limited variations in the elements throughout their lives, the profiles of which were confined in small areas at very low Sr and Ba levels. Profiles for the Mun River, Tonle Sap and MM fish were similar to each other and generally increased both in Sr and Ba with fish growth.

**Figure 6:**
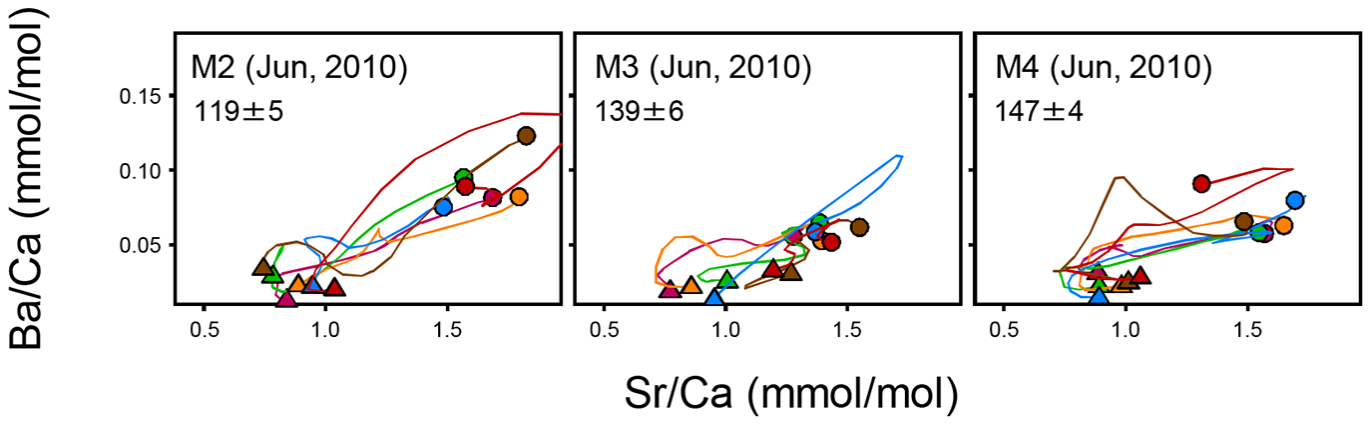
Sr-Ba elemental profiles along *H. lobatus* otolith transect. Triangle: otolith core. Circle: otolith surface. All samples were collected in June 2010 from 3 sites in the Mekong mainstream. Within a given site, different colors denote different individuals.

Among three sampling sites along MM (M2 below Khone Falls, and M3 and M4 above the falls), the otolith surface signatures of *H. lobatus* were different (Pillai's trace  = 0.811, *F*_4, 28_ = 4.779, p = 0.005), but their core signatures were not (Pillai's trace  = 0.190, *F*_4, 28_ = 0.733, p>0.05, Fig. 6). Between the two species both collected at M2 in June 2010 (Figs. 5 & 6), otolith surface signatures were not different (Pillai's trace  = 0.459, *F*_2, 9_ = 3.824, p>0.05), but core signatures were different (Pillai's trace  = 0.536, *F*_2, 9_ = 5.196, p = 0.032). Most profiles in both species commonly exhibited a slight decrease (ca. 0.2 mmol/mol) in Sr/Ca immediately outside of primordia at a very early life stage of fish, which was followed by increases in Sr/Ca and Br/Ca at later life stages.

## Discussion

### Uptake of elements by Siamese mud carp otoliths

The Siamese mud carp undertook complex migrations as revealed by Sr-Ba profiles along the otolith growth axis. There were similarities in the profiles among individuals within regions and dissimilarities between regions, suggesting that fish from a given region hatched out in a similar environment and migrated in schools before being harvested by fishermen, but that their natal origins and migration pathways were different across most regions throughout the Mekong basin.

Similarly to many previous studies, strontium was the most reliable marker element followed by barium to elucidate Siamese mud carp migrations in the Mekong River [26], [30], [41], [42]. Strontium partition coefficients for *H. siamensis* (0.409) and *H. lobatus* (0.496) were comparable to those observed for freshwater fishes in temperate and arctic climates such as cutthroat trout *Oncorhynchus clarki lewisi* (0.40) [26] and Arctic grayling *Thymallus arcticus* (0.369) [30]. Barium partition coefficient for *H. siamensis* (0.055) was again similar to those for cutthroat trout (0.04) and Arctic grayling (0.051). Magnesium incorporation to otoliths was not significantly correlated with water Mg/Ca. However, the negative, although insignificant, relationship between water and otolith Mg we found in *H. siamensis* was also observed in the otoliths (and scales) of cutthroat trout [26], for which an underlying mechanism has yet to be understood. We found no correlation between water and otolith Mn, which was similar to the findings in previous studies [41]–[44]. Although a mechanism for Mn incorporation into otoliths is largely unknown, physiological processes such as dietary uptake have been implicated [42], [43].

A temperature effect on otolith microchemistry is generally less important than ambient elemental concentration and depends on the ranges of both temperatures and elemental concentrations [44]–[47]. Mugiya and Tanaka [48] observed a positive influence of water temperature on otolith Sr uptake in goldfish (cyprinid), but it was on the order of one fifth of Sr/Ca in ambient water. Miller [47] observed a positive temperature effect on Sr (and Mg) uptake in juvenile Chinook salmon *Oncorhynchus tshawytscha* otoliths, but the effect was observed in the Sr-rich saline environment and not in fresh water.

### Migrations by region

Despite some limitations of and uncertainties about the technique, we could detect region-specific as well as common basin-wide patterns in the migrations of Siamese mud carp. In the Thai North, *H. siamensis* exhibited greater variability in Ba rather than Sr throughout their life history, resulting in a peculiar shape in the elemental profiles. This may be explained by a difference in geological structure between upper and middle-to-lower Mekong basins. The upper basin including the Thai North is characterized by multiple tectonic plates that are intricately separated by several major Tertiary-Quaternary sedimentary basins, whereas the middle Mekong basin, especially the vast area called the Khorat Plateau, encompasses three regions, i.e., Songkhram, Gam and Mun river basins in northeastern Thailand, and is covered uniformly by Cretaceous sedimentary rocks [7], [49]. Therefore, the larger otolith Ba variability does not necessarily translate into longer fish migrations but reflects a greater spatial complexity in water elements encountered by the fish. Due to the unique otolith elemental profiles, *H. siamensis* from the Thai North likely did not share migration routes with individuals from the other regions, corroborating a finding by Adamson et al. [23] who identified a genetically distinct population of the species in this region.

Otolith core as well as surface elemental signatures were not different between the Songkhram and Mun Rivers. Adamson et al. [23] considered *H. siamensis* from these regions as a single genetically homogeneous stock. Although both the mean otolith chemical signatures and the genetic analysis could not differentiate between the two regions, the shape of the otolith elemental profiles was considerably different with much more diverse core signatures for the Songkhram fish in the dry season than the Mun fish, suggesting that multiple *H. siamensis* stocks utilize the Songkhram River in this season. Located between the Songkhram and Mun Rivers, the Gam River had a *H. siamensis* population with extremely low levels of, and a limited variability, in otolith Sr and Ba during their life history. This will be discussed later in the context of a possible damming influence on this population. Elemental profiles largely overlapped between Mun, Tonle Sap and MM with no significant differences in Sr or Ba at both otolith core and surface. However, because the genetic analysis identified a single stock of *H. siamensis* below the Khone Falls [23], *H. siamensis* in the Mun River, which connects with MM above the Khone Falls, likely have a different natal origin from fish in Tonle Sap or MM. During the peak flood season, the Mekong River water causes the Tonle Sap River to reverse its flow [50], likely mixing the water and homogenizing elemental signatures throughout the vast geographical extent. This unique hydrological event may have contributed to the similar otolith elemental profiles between the Tonle Sap and MM regions. However, it does not preclude the possibility of the species collected in MM originating from, or spending part of their life in, the Tonle Sap Lake [11], [12].

*H. lobatus* collected at M2 (ca. 70 km below the Khone Falls), M3 (right above the falls), and M4 (ca. 130 km above the falls) exhibited fairly synchronized indistinguishable Sr-Ba profiles with the core elemental signatures being statistically not different among the sites. This finding supports the previous notion that, unlike *H. siamensis*, *H. lobatus* can traverse the Khone Falls [12] and that no genetic differentiation exists among individuals collected below and above the waterfalls [22]. Although the similarity in the otolith profiles and particularly core elemental signatures could be explained by the passive drift of *H. lobatus* from above the Khone Falls downstream to M2 below the waterfalls, Hurwood et al. [22] considers it unlikely because the haplotypic diversity of the species was higher above the Khone Falls than below the falls, indicating the active upstream dispersal of the species over the waterfalls. In summary, the Khone Falls are probably not a migration barrier at least to *H. lobatus* as local ecological knowledge [4], catch statistics [12], genetic analysis [22] and otolith microchemistry have all indicated.

Otolith elemental profiles of the two species commonly exhibited a sharp drop in Sr level just outside of a primordium at a very early life stage. It spanned approximately 200–300 µm of an otolith distance from the core or equivalently 15–20% of the average fish life as an average otolith core-edge length was 1,440 µm. Although it only takes 12–16 hrs for Siamese mud carp to hatch out after spawning [51], it takes seven days for their fin rays to start developing and 35–40 days to fully develop [52]. During this early developmental stage, they would be flushed downstream by water currents for long distances before becoming fully capable of swimming against river flows. However, relating the initial drops in otolith Sr to passive movement of fish with water currents may not be a straightforward explanation because water Sr/Ca does not necessarily decrease in a longitudinal, downstream direction in the Mekong River and its tributaries [53]. Nothing is known about finer-scale lateral variations in Sr/Ca across a river channel and floodplain where the species are believed to rear, so that the passive drift of fish eggs and larvae cannot be entirely ruled out as the possible explanation of the phenomenon. Alternatively, it could have resulted from reduced Sr uptake by the otoliths of Siamese mud carp larvae at the onset of the rainy season when water temperatures start to drop. An annual temperature variation is roughly eight degrees in Celsius in the Mekong tributaries (M. Fukushima, unpublished data), which is substantial for tropical ecosystems. Despite relatively minor influence of temperature on otolith elemental uptake as described above, a rapid decrease in water temperature during the early developmental stage may have played a role.

We found that Zn and Ba had insignificant correlations between water and otolith surface during the dry season. It may indicate more active, faster migration by the species in this season, as fish would spend less time at any given location for elements unique to that location to be incorporated into otoliths. Otolith trace elements such as Sr and Ba change rapidly in response to changes in water chemistry, but it typically takes about two weeks for the otolith concentrations to stabilize and fully reflect ambient concentrations [48], [54], [55].

### Effects of damming on Siamese mud carp populations

The migration of *H. siamensis* was severely suppressed in the Gam River. Otolith profiles for this region not only occupied a small confined area in the Sr-Ba biplots but also overlapped minimally with profiles for the other regions including MM. Fish sampling sites in the Gam River were located in-between irrigation dams that must have severely impeded fish movement within the tributary as well as movement to MM. Although a fishladder has been installed at each dam site in this river, its performance has received a low rating because of its limited efficacy favoring only small-sized short to medium-distance fish migrants [36]. *H. siamensis* are known to be able to establish populations in a lentic system such as a reservoir [56] and even river reaches between barriers [4] as they probably do in the Gam River judging from the large body size of individuals collected. Because of this flexibility, the species has been among the candidates for fish stocking to enhance fisheries production in reservoirs in Thailand and Laos [57]. However, their gonadal as well as somatic growth is significantly slower than those observed in wild populations in lotic systems [56], [58], and therefore they do not flourish in those man-made water bodies [59], [60].

In contrast, *H. siamensis* migration appeared not to be suppressed in the Mun River despite a possible impediment by the Pak Mun dam near the confluence of the Mekong River. Water Sr-Ba signatures in the Mun River and MM were relatively constant throughout a year and across years within the regions, and they were fairly distinct from each other. Nevertheless, the otolith Sr-Ba profiles of the two regions considerably varied and overlapped, suggesting that the Mun fish had migrated as extensively as did MM fish through the sluice gates of the Pak Mun hydroelectric dam, presumably from MM [58]. This study reaffirmed the importance of opening the sluice gates during the flood season to conserve fish species diversity and local fisheries [37], [38].

*H. lobatus* will suffer from the planned Don Sahong hydropower project in the middle of Hoo Sahong channel, the single most important fish migration corridor bypassing the Khone Falls. Otolith Sr-Ba profiles indicated little difference in the natal origin and subsequent migration pathways of the fish collected below and above Khone Falls. Along with *H. lobatus*, other commercially important fish species would be equally affected by this dam. *Pangasius krempfi*, highly valued large-bodied fish species mostly harvested around the Khone Falls area during their spawning migration, were recently confirmed by otolith microchemistry to be anadromous, down-migrating to the sea for feeding and up-migrating the Mekong River over the Khone Falls for reproduction [61]. Artificial fish passes are unlikely to be an effective mitigation measure in the Mekong River because of the size of fish migrations which can reach as much as 30 tonnes per hour [62] and because of the significant number of migratory fish species that differ in body size and shape and behavior [5].

The impact of this and many other hydropower projects proposed in the Mekong basin on Siamese mud carp populations can be devastating in the light of the findings of this study. First, the species have discrete and rigid migration pathways that are consistent among individuals within a region but different among regions, making it unlikely that fish seek alternative migration routes once a dam blocks their original migration route. Second, their migration range has already been severely suppressed in a dammed tributary of the Mekong, i.e., the Gam River, despite the existence of fish passes. Third, there is a population of *H. lobatus* distributed along the >200-km stretch of the Mekong mainstream that are migrating over the Khone Falls, most likely through the Hoo Sahong channel where the Don Sahong dam is planned.

## Supporting Information

Table S1 **Elemental ratios of the 158 water samples collected from the lower Mekong basin during 2007-2010.**
(XLSX)

Table S2 **Fork length (mean ± SD) and sample size (n) of Siamese mud carp (** ***Henicorhynchus siamensis*** **and** ***H. lobatus*** **) sampled by month and region.**
(DOCX)

## References

[1] Barthem R, Goulding M (1997) The catfish connection: ecology, migration, and conservation of Amazon predators. Columbia University Press. 144 p.

[2] Northcote TG (1998) Migratory behavior of fish and its significance to movement through riverine fish passage facilities. In: Jungwirth M, Schmutz S, Weiss S, editors. Fish migration and fish bypasses. Fishing News Books. Oxford, UK, Blackwell Science Ltd. Publisher.pp. 3–18.

[3] Welcomme RL (2001) Inland fisheries ecology and management. Fishing News Books, Oxford, UK. 358 p.

[4] Poulsen AF, Hortle KG, Valbo-Jorgensen J, Chan S, Chhuon CK, et al.. (2004) Distribution and ecology of some important riverine fish species of the Mekong River basin. MRC Technical Paper No. 10. Mekong River Commission, Phnom Penh. 116 p.

[5] Baran E (2010) Mekong fisheries and mainstream dams. Fisheries sections. In: Mekong River Commission strategic environmental assessment of hydropower on the Mekong mainstream, International Centre for Environmental Management, Hanoi, Viet Nam. 145 p.

[6] Hortle KG (2009) Fisheries of the Mekong River basin. In: Campbell IC, editor. The Mekong biophysical environment of an international river basin. Academic Press. pp. 197–250.

[7] Rainboth WJ (1996) Fishes of the Cambodian Mekong. FAO, Rome. 265 p.

[8] Baran E (2006) Fish migration triggers in the Lower Mekong Basin and other tropical freshwater systems. MRC Technical Paper No. 14. Mekong River Commission, Vientiane.56 p.

[9] Van Zalinge N, Degen P, Chumnarn P, Sam N, Jensen J, et al.. (2004) The Mekong River system. In: Welcomme RL, Petr T. editors. Proceedings of the second international symposium on the management of large rivers for fisheries, Vol. 1. FAO, Bangkok. pp. 333–355.

[10] Barlow C, Baran E, Halls AS, Kshatriya M (2008) How much of the Mekong fish catch is at risk from mainstream dam development? Catch and Culture 14: 16–21.

[11] Lieng S, Yim C, van Zalinge NP (1995) Freshwater fisheries of Cambodia: the bagnet (*Dai*) fishery in the Tonle Sap River. Asian Fisheries Science 8: 255–262.

[12] Baird IG, Flaherty MS, Phylavanh B (2003) Rhythms of the river: lunar phases and migrations of small carps (Cyprinidae) in the Mekong River. Natural History Bulletin of the Siam Society 51: 5–36.

[13] Baran E, Baird IG, Cans G (2005) Fisheries bioecology at the Khone Falls (Mekong River, Southern Laos). WorldFish Center. 84 p.

[14] Nguyen T, Yen H, Sunada K, Oishi S, Ikejima K, et al (2009) Stock assessment and fishery management of *Henicorhynchus* spp., *Cyclocheilichthys enoplos* and *Channa micropeltes* in Tonle Sap Great Lake, Cambodia. Journal of Great Lakes Research 35: 169–174.

[15] Dugan PJ, Barlow C, Agostinho AA, Baran E, Cada GF, et al (2010) Fish migration, dams, and loss of ecosystem services in the Mekong basin. Ambio 39: 344–348. 20799685 10.1007/s13280-010-0036-1 PMC3357701

[16] Jackson D, Marmulla G (2001) The influence of dams on river fisheries. In: Marmulla G, editor. Dams, fish and fisheries. Opportunities, challenges and conflict resolution. FAO Fisheries technical paper No. 419, Rome. pp. 1–44.

[17] Bunn SE, Arthington AH (2002) Basic principles and ecological consequences of altered flow regimes for aquatic biodiversity. Environmental Management 30: 492–507. 12481916 10.1007/s00267-002-2737-0

[18] Ferguson JW, Healey M, Dugan P, Barlow C (2011) Potential effects of dams on migratory fish in the Mekong River: lessons from salmon in the Fraser and Columbia Rivers. Environmental Management 47: 141–159. 20924582 10.1007/s00267-010-9563-6

[19] Roberts TR (1997) Systematic revision of the tropical Asian labeon cyprinid fish genus *Cirrhinus*, with descriptions of new species and biological observations on *C. lobatus* . Natural History Bulletin of the Siam Society 45: 171–203.

[20] Roberts TR, Baird IG (1995) Traditional fisheries and fish ecology on the Mekong River at Khone Waterfalls in southern Laos. Natural History Bulletin of the Siam Society 43: 219–262.

[21] Suntornratana U, Poulsen A, Visser T, Nakkeaw S, Talerkkeatleela T (2002) Migration onto the floodplain of the Songkhram River Basin. In: Proceedings of the 4th Technical Symposium on Mekong Fisheries. MRC Conference Series, No. 2. Mekong River Commission, Phnom Penh. pp. 270–282.

[22] Hurwood DA, Adamson EAS, Mather PB (2008) Evidence for strong genetic structure in a regionally important, highly vagile cyprinid (*Henicorhynchus lobatus*) in the Mekong River Basin. Ecology of Freshwater Fish 17: 272–283.

[23] Adamson EAS, Hurwood DA, Baker AM, Mather PB (2009) Population subdivision in Siamese mud carp *Henicorhynchus siamensis* in the Mekong River basin: implications for management. Journal of Fish Biology 75: 1371–1392. 20738620 10.1111/j.1095-8649.2009.02369.x

[24] Campana SE (1999) Chemistry and composition of fish otoliths: pathways, mechanisms and applications. Marine Ecology Progress Series 188: 263–297.

[25] Elsdon TS, Wells BK, Campana SE, Gillanders BM, Jones CM, et al (2008) Otolith chemistry to describe movements and life-history parameters of fishes: hypotheses, assumptions, limitations and inferences. Oceanography and Marine Biology: An Annual Review 46: 297–330.

[26] Wells BK, Rieman BE, Clayton JL, Horan DL, Jones CM (2003) Relationships between water, otolith, and scale chemistries of westslope cutthroat trout from the Coeur d'Alene River, Idaho: the potential application of hard-part chemistry to describe movements in freshwater. Transactions of the American Fisheries Society 132: 409–424.

[27] Muhlfeld CC, Marotz B, Thorrold SR, FitzGerald JL (2005) Geochemical signatures in scales record stream of origin in westslope cutthroat trout. Transactions of the American Fisheries Society 134: 945–959.

[28] Crook DA, Gillanders BM (2006) Use of otolith chemical signatures to estimate carp recruitment sources in the mid-Murray River, Australia. River Research and Applications 22: 871–879.

[29] Zimmerman CE, Edwards GW, Perry K (2009) Maternal origin and migratory history of steelhead and rainbow trout captured in rivers of the Central Valley, California. Transactions of the American Fisheries Society 138: 280–191.

[30] Clarke AD, Telmer KH, Shrimpton JM (2007) Habitat use and movement patterns for a fluvial species, the Arctic grayling, in a watershed impacted by a large reservoir: evidence from otolith microchemistry. Journal of Applied Ecology 44: 1156–1165.

[31] Pangle KL, Ludsin SA, Fryer BJ (2010) Otolith microchemistry as a stock identification tool for freshwater fishes: testing its limits in Lake Erie. Canadian Journal of Fisheries and Aquatic Sciences 67: 1475–1489.

[32] Muhlfeld CC, Thorrold SR, McMahon TE, Marotz B (2012) Estimating westslope cutthroat trout (*Oncorhynchus clarkii lewisi*) movements in a river network using strontium isoscapes. Canadian Journal of Fisheries and Aquatic Sciences 69: 906–915.

[33] Coghlan SM Jr, Lyerly MS, Bly TR, Williams JS, Bowman D, et al (2007) Otolith chemistry discriminates among hatchery-reared and tributary-spawned salmonines in a tailwater system. North American Journal of Fisheries Management 27: 531–541.

[34] Halden NM, Friedrich LA (2008) Trace element distributions in fish otoliths: natural markers of life histories, environmental conditions and exposure to tailings effluence. Mineralogical Magazine 72: 591–603.

[35] Baird IG (1996) Khone Falls fishers. Catch and Culture: Mekong Fisheries Network Newsletter 2: 2–3.

[36] Pongsri C, Thongpan W, Sricharoendham B, Ngoichansri S, Suwanpeng N (2008) Assessment of upstream migration via fish ladders in the Nam Gam River for fish ladder and fisheries resources management. Technical Paper No. 10. Department of Fisheries, Bangkok. [in Thai].

[37] Jutagate T, Krudpan C, Ngamsnae P, Lamkom T, Payooha K (2005) Changes in the fish catches during a trial opening of sluice gates on a run-of-the river reservoir in Thailand. Fisheries Management and Ecology 12: 57–62.

[38] Jutagate T, Thapanand T, Tabthipwan P (2007) Is sluice gate management beneficial for spawning migrations? The case of the shark catfish (*Helicophagus waandersii*) in the Mun River below the Pak Mun Dam, Thailand. River Research and Applications 23: 87–97.

[39] Hoff GR, Logan DJ, Markle DF (1997) Otolith morphology and increment validation in young Lost River and shortnose suckers. Transactions of the American Fisheries Society 126: 488–494.

[40] Longerich HP, Jackson SE, Günther D (1996) Laser ablation-inductively coupled plasma-mass spectrometric transient signal data acquisition and analyte concentration calculation. J Analyt Atom Spectrom 11: 899–904.

[41] Elsdon TS, Gillanders BM (2003) Relationship between water and otolith elemental concentrations in juvenile black bream *Acanthopagrus butcheri* . Marine Ecology Progress Series 260: 263–272.

[42] Gibson-Reinemer DK, Johnson BM, Martinez PJ, Winkelman DL, Koenig AE, et al (2009) Elemental signatures in otoliths of hatchery rainbow trout (*Oncorhynchus mykiss*): distinctiveness and utility for detecting origins and movement. Canadian Journal of Fisheries and Aquatic Sciences 66: 513–524.

[43] Sanchez-Jerez P, Gillanders BM, Kingstord MJ (2002) Spatiala variability of trace elements in fish otoliths: comparison with dietary items and habitat constituents in seagrass meadows. Journal of Fish Biology 61: 801–821.

[44] Collingsworth PD, Van Tassell JJ, Olesik JW, Marshall EA (2010) Effects of temperature and elemental concentration on the chemical composition of juvenile yellow perch (*Perca flavescens*) otoliths. Canadian Journal of Fisheries and Aquatic Sciences 67: 1187–1196.

[45] Secor DH, Rooker JR (2000) Is otolith strontium a useful scalar of life cycles in estuarine fishes? Fisheries Research 46: 359–371.

[46] Martin GB, Thorrold SR, Jones CM (2004) Temperature and salinity effects on strontium incorporation in otoliths of larval spot (*Leiostomus xanthurus*). Canadian Journal of Fisheries and Aquatic Sciences 61: 34–42.

[47] Miller JA (2011) Effects of water temperature and barium concentration on otolith composition along a salinity gradient: implications for migratory reconstructions. Journal of Experimental Marine Biology and Ecology 405: 42–52.

[48] Mugiya T, Tanaka S (1995) Incorporation of water-borne strontium into otoliths and its turnover in the goldfish *Carassius auratus*: effects of strontium concentrations, temperature, and 17-beta-estradiol. Fisheries Science 61: 29–35.

[49] Wakita K, Okubo Y, Bandibas JC, Lei X, Schulte MJD, et al.. (2004) Digital Geologic Map of East and Southeast Asia 1:2,000,000 Second Edition. Geological Survey of Japan, AIST.

[50] Campbell IC, Say S, Beardall J (2009) Tonle Sap Lake, the Heart of the Lower Mekong. In: Campbell IC, editor. The Mekong – biophysical environment of an international river basin. Academic Press. pp. 251–272.

[51] Puangchareon S, Meksumpun C (2006) Spawning season and area of Jullien's mud carp (*Henicorhynchus siamensis* de Beaufort, 1927) in Pasak Jolasid Reservoir, Lop Buri Province. In: Proceedings of 44th Kasetsart University Annual Conference: Fisheries. pp. 21–28. [in Thai].

[52] Termvidchakorn A, Hortle KG (2013) A guide to larvae and juveniles of some common fish species from the Mekong River Basin. MRC Technical Paper No. 38. Mekong River Commission, Phnom Penh. 234 p.

[53] Fukushima M, Nohara S (2012) Environmental impact assessment of dam development in the Mekong River. Journal of Japan Society on Water Environment. 35: 53–58. [in Japanese].

[54] Milton DA, Chenery SR (2001) Sources and uptake of trace metals in otoliths of juvenile barramundi (*Lates calcarifer*). Journal of Experimental Marine Biology and Ecology 264: 47–65.

[55] Hale R, Swearer SE (2008) Otolith microstructural and microchemical changes associated with settlement in the diadromous fish *Galaxias maculatus* . Marine Ecology Progress Series 354: 229–234.

[56] Suvarnaraksha A, Lek S, Lek-Ang S, Jutagate T (2011) Life history of the riverine cyprinid *Henicorhynchus siamensis* (Sauvage, 1881) in a small reservoir. Journal of Applied Ichthyology 27: 995–1000.

[57] Jutagate T (2009) Reservoir fisheries of Thailand. In: De Silva SS, Amarasinghe US, editors. Status of reservoir fisheries in five Asian countries. Network of Aquaculture Centers in Asia-Pacific, Bangkok. pp. 96–113.

[58] Phomikong P, Fukushima M, Sricharoendham B, Nohara S, Jutagate T (2014) Diversity and community structure of fishes in the regulated vs. unregulated tributaries of the Mekong River. River Research and Applications (In press).

[59] Lamberts D (2001) Tonle Sap fisheries: A case study on floodplain gillnet fisheries. FAO Regional Office for Asia-Pacific Publication No. 2001 / 11. FAO, Bangkok. 133 p.

[60] Chheng P, Touch BT, Baran E, Vann LS (2005) Biological review of important Cambodian fish species, based on FishBase 2004. WorldFish Center, Phnom Penh. 127 p.

[61] Hogan Z, Baird IG, Radtke R, Vander Zanden MJ (2007) Long distance migration and marine habitation in the tropical Asian catfish, *Pangasius krempfi* . Journal of Fish Biology 71: 818–832.

[62] Baran E, Van Zalinge N, Ngor Peng Bun, Baird IG, Coates D (2001) Fish resource and hydrobiological modelling approaches in the Mekong Basin. ICLARM, Penang, Malaysia and the Mekong River Commission Secretariat, Phnom Penh, Cambodia. 62 p.

