# Supplementary material for: Potential Effects of Hydroelectric Dam Development in the Mekong River Basin on the Migration of Siamese Mud Carp (Henicorhynchus siamensis and H. lobatus) Elucidated by Otolith Microchemistry
Source: PLoS One. 2014 Aug 6;9(8):e103722. doi: 10.1371/journal.pone.0103722 (PMC4123893; doi:10.1371/journal.pone.0103722)
Supplement: Table S2 — Fork length (mean ± SD) and sample size (n) of Siamese mud carp ( Henicorhynchus siamensis and H. lobatus ) sampled by month and region. (DOCX) [file pone.0103722.s002.docx]

**Table S2. Fork length (mean ± SD) and sample size (*n*) of Siamese mud carp (*Henicorhynchus siamensis* and *H. lobatus*) sampled by month and region.**

|  |  |  | **Fish size (mm)** | |  |
| --- | --- | --- | --- | --- | --- |
| **Year/Month** | **Region** | **Site** | **Mean** | **SD** | ***n*** |
| *Henicorhynchus siamensis* | |  |  |  |  |
| **2007** |  |  |  |  |  |
| March | Mun | U8 | 193 | - | 1 |
| May | Mun | U7 | 130 | 3 | 2 |
| November | Thai North | N1 | 165 | 6 | 5 |
| November | Thai North | N2 | 121 | 13 | 5 |
| November | Mun | U3 | - | - | 5 |
| **2008** |  |  |  |  |  |
| February | Mun | U6 | 134 | 8 | 4 |
| March | Mun | U6 | 139 | 11 | 5 |
| March | Mun | U5 | 136 | 5 | 2 |
| May | Xekong | X1 | 157 | - | 1 |
| May | MM | M2 | 123 | 5 | 5 |
| May | MM | M3 | 143 | - | 1 |
| July | Mun | U3 | 152 | 42 | 2 |
| July | Mun | U5 | 136 | 9 | 3 |
| July | Xekong | X2 | 179 | 3 | 2 |
| November | Songkhram | S1 | 99 | 4 | 6 |
| **2009** |  |  |  |  |  |
| April | Tonle Sap | T5 | 115 | 6 | 5 |
| April | Tonle Sap | T2 | 121 | 5 | 3 |
| April | Tonle Sap | T1 | 118 | 9 | 3 |
| April | Tonle Sap | T3 | 137 | 6 | 6 |
| April | Tonle Sap | T4 | 133 | 5 | 5 |
| August | Songkhram | S2 | 107 | 4 | 2 |
| August | Songkhram | S4 | 127 | - | 1 |
| August | Gam | G4 | 181 | - | 1 |
| August | Mun | U2 | 90 | 12 | 2 |
| August | Mun | U4 | 112 | 3 | 3 |
| October | Songkhram | S2 | 98 | 29 | 2 |
| October | Gam | G1 | 91 | - | 1 |
| October | Gam | G4 | 92 | - | 1 |
| October | Mun | U2 | 108 | 0 | 2 |
| October | Mun | U4 | 117 | 14 | 2 |
| December | Songkhram | S2 | 137 | 3 | 4 |
| December | Gam | G2 | 128 | 14 | 4 |
| December | Mun | U1 | 69 | 6 | 2 |
| **2010** |  |  |  |  |  |
| February | Songkhram | S2 | 142 | 5 | 5 |
| February | Gam | G2 | 131 | 7 | 5 |
| March | MM | M1 | 116 | 5 | 7 |
| March | MM | M2 | 121 | 3 | 8 |
| April | Songkhram | S2 | 113 | 9 | 5 |
| April | Songkhram | S3 | 122 | 13 | 3 |
| April | Gam | G3 | 138 | 4 | 7 |
| April | Mun | U4 | 156 | 8 | 13 |
| June | Songkhram | S3 | 136 | 5 | 5 |
| June | Songkhram | S4 | 148 | 11 | 4 |
| June | Gam | G4 | 106 | 39 | 2 |
| June | Mun | U4 | 153 | 8 | 5 |
| June | MM | M2 | 133 | 4 | 6 |
| *H. lobatus* |  |  |  |  |  |
| **2008** |  |  |  |  |  |
| May | Xekong | X1 | 118 | - | 1 |
| May | MM | M2 | 102 | 8 | 2 |
| November | Xekong | X2 | 125 | 10 | 4 |
| **2010** |  |  |  |  |  |
| June | MM | M2 | 118 | 5 | 6 |
| June | MM | M3 | 138 | 6 | 6 |
| June | MM | M4 | 146 | 4 | 5 |
